# Convergent production and tolerance among 107 woody species and divergent production between shrubs and trees

**DOI:** 10.1038/srep20485

**Published:** 2016-02-08

**Authors:** Wei-Ming He, Zhen-Kai Sun

**Affiliations:** 1 State Key Laboratory of Vegetation and Environmental Change, Institute of Botany, Chinese Academy of Sciences, Haidian District, Beijing 100093, China

## Abstract

Green leaves face two fundamental challenges (i.e., carbon fixation and stress tolerance) during their lifespan. However, the relationships between leaf production potential and leaf tolerance potential have not been explicitly tested with a broad range of plant species in the same environment. To do so, we conducted a field investigation based on 107 woody plants grown in a common garden and complementary laboratory measurements. The values, as measured by a chlorophyll meter, were significantly related to the direct measurements of chlorophyll content on a leaf area basis. Area-based chlorophyll content was positively correlated with root surface area, whole-plant biomass, leaf mass per area (LMA), and force to punch. Additionally, LMA had a positive correlation with force to punch. Shrubs had a higher leaf chlorophyll content than trees; however, shrubs and trees exhibited a similar leaf lifespan, force to punch, and LMA. These findings suggest that the production potential of leaves and their tolerance to stresses may be convergent in woody species and that the leaf production potential may differ between shrubs and trees. This study highlights the possibility that functional convergence and divergence might be linked to long-term selection pressures and genetic constraints.

Leaf traits of plants can control key ecological functions. For example, the carbon economy of leaves underlies their biomass production^1^,^2^; the structure of a leaf determines its tolerance to stresses, including drought, herbivory, light and temperature^3^,^4^; and leaf chemistry governs carbon and nutrient cycling^5^,^6^. Accordingly, the leaf economics spectrum has received increasing attention since the 1990s. For example, plant species from the tropics to the tundra exhibit convergence in leaf functioning^7^; fast-return species are characterized by having low a LMA, a short leaf lifespan, and high leaf nitrogen (N), phosphorus (P), photosynthesis and respiration^8^; tropical leaves are not mechanically more resistant than temperate leaves^9^; and the leaf economics spectrum is approximately distributed proportional to leaf area instead of mass^10^.

Studying the leaf economics spectrum has contributed much to our understanding of ecological functions at fine and broad scales^2^,^7^,^8^,^9^,^10^,^11^,^12^,^13^,^14^. Actually, the leaf economics spectrum reflects multiple signals from environments, phylogeny, natural selection, or even sampling. To date, few if any studies have explicitly tested the generality of the leaf economics spectrum in the context of the same environments. Common gardens provide an ideal platform for testing this generality because striking differences in climate, soils, and sampling can be eliminated and the legacies of evolution and selection can be highlighted. According to the worldwide leaf economics spectrum^7^,^8^, trade-offs between leaf production and leaf stress tolerance seem to occur among a broad range of plant species or between functional types. To test the generality of this prediction, we selected 107 woody species grown in a common garden for over 20 years and categorized them into shrubs and trees (i.e., two different functional types).

Here, we selected four leaf traits (i.e., LMA, force to punch, leaf chlorophyll, and leaf lifespan) as our focal traits for the following reasons. LMA and force to punch are two key structural/mechanical traits that determine the potential of plants to tolerate multiple stresses (e.g., drought, herbivory, light, and temperature)^9^,^15^,^16^. Leaf chlorophyll is linked to the production potential of plants^17^, and leaf lifespan reflects the duration of the ability of leaves to fix carbon^8^. We addressed two central questions: the relationship between leaf production potential and leaf stress tolerance potential either across 107 woody species or between functional types. Additionally, we answered two secondary questions: (1) whether the value measured by a portable SPAD-502 chlorophyll meter (SPAD: soil and plant analysis development; hereafter referred to as SPAD values) can indicate chlorophyll content and (2) whether chlorophyll content is positively correlated with root area and biomass production.

## Results

### SPAD values versus direct measurements of chlorophyll

**Figure 1:**
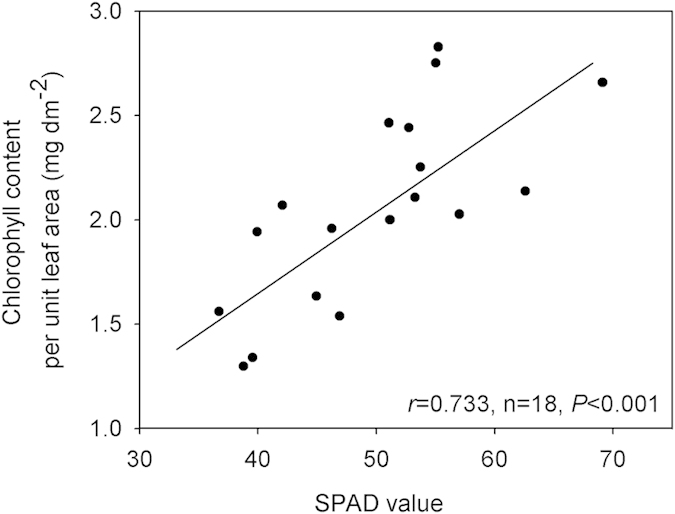
Relation between SPAD values and the directly measured chlorophyll content per unit leaf area. Each filled circle represents the values for a given species.

Leaf chlorophyll content, measured directly on the basis of leaf area, significantly increased with SPAD values for shrubs (*r* = 0.698, n = 9, *P* = 0.027) and trees (*r* = 0.7903, n = 9, *P* = 0.011). Figure 1 presents the relationship between SPAD values and chlorophyll content per unit leaf area across 18 woody species (*r* = 0.733, *P* < 0.001). Thus, SPAD values effectively indicated leaf chlorophyll content per unit leaf area.

### SPAD values, root area, and biomass production

**Figure 2:**
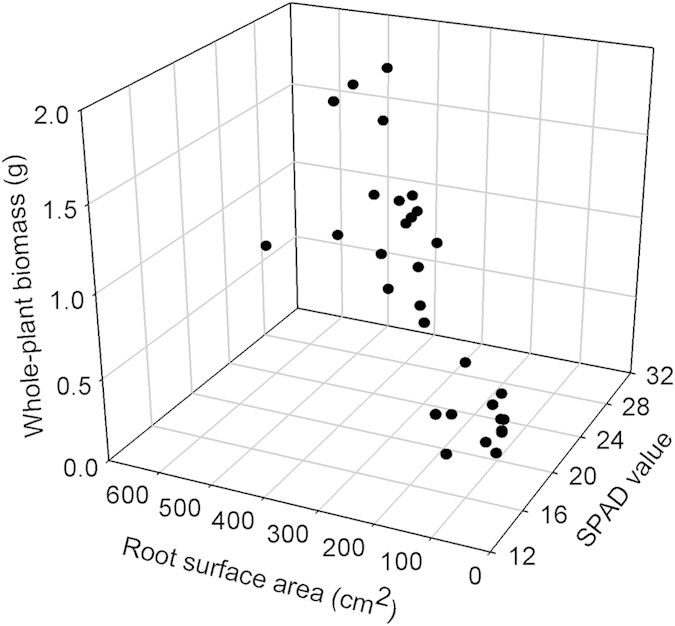
Three-way trait relationships among SPAD values, root surface area, and whole-plant biomass. Each filled circle represents the values for a given individual.

There was a positive correlation between SPAD values and root surface area (Fig. 2: *r* = 0.821, n = 30, *P* < 0.001). The whole-plant biomass of plants significantly increased with their SPAD values (Fig. 2: *r* = 0.913, n = 30, *P* < 0.001) and root surface area (Fig. 2: *r* = 0.888, n = 30, *P* < 0.001). Accordingly, individuals with high SPAD values and root area had a high potential to yield biomass.

### Leaf traits across woody species

There were 1.8-, 3.0-, 5.4- and 1.8-fold variations in chlorophyll, LMA, force to punch, and leaf lifespan, respectively. These results show that different leaf traits had different levels of variation (i.e., force to punch > LMA > chlorophyll = leaf lifespan), implying that the inherent determinants underlying these variations may differ, depending on trait identity.

**Figure 3:**
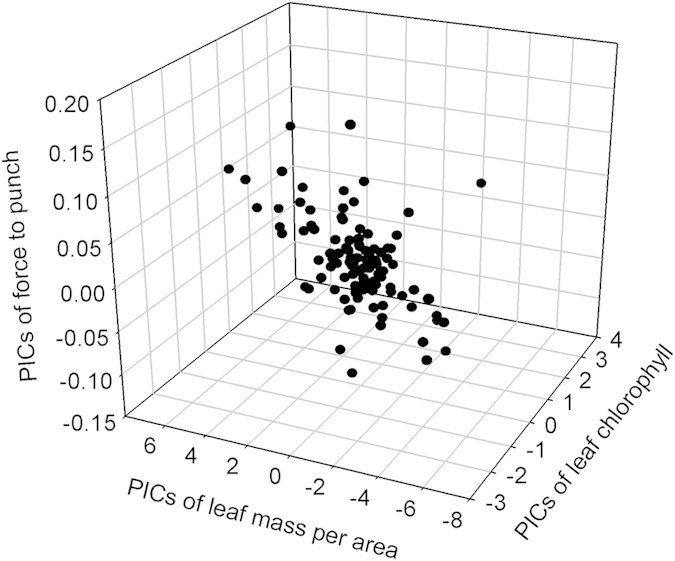
Three-way traits relationships among PICs of leaf chlorophyll, PICs of leaf mass per area, and PICs of force to punch. PICs: phylogenetic independent contrasts. Each filled circle represents the values for a given species.

The following results were presented in the form of phylogenetic independent contrasts (PICs). Leaf lifespan was not correlated to chlorophyll content (*r* = 0.147, n = 49, *P* = 0.156) and LMA (*r* = 0.009, n = 49, *P* = 0.475). LMA increased with chlorophyll content (Fig. 3; *r* = 0.509, n = 107, *P* < 0.001), force to punch increased with chlorophyll content (Fig. 3; *r* = 0.289, n = 107, *P* = 0.022), and force to punch was positively associated with LMA (Fig. 3; *r* = 0.532, n = 107, *P* < 0.001). Accordingly, variable chlorophyll content had differential effects on force to punch, LMA, and leaf lifespan, and changing LMA had contrasting effects on force to punch and leaf lifespan.

### Leaf traits between functional types

**Figure 4:**
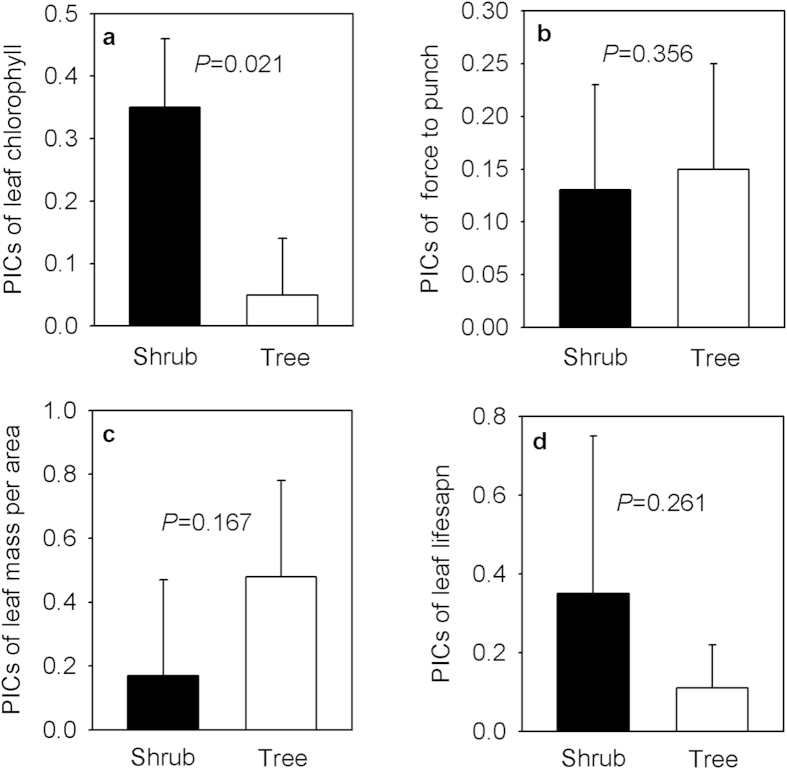
Comparisons of PICs of leaf chlorophyll. (**a**), PICs of force to punch (**b**), PICs of leaf mass per area (**c**), and PICs of leaf lifespan (**d**) between shrubs and trees. PICs: phylogenetic independent contrasts. The data are expressed as the mean + 1 SE.

The PICs of leaf chlorophyll content were significantly greater in shrubs than in trees (Fig. 4a; *P* = 0.021), and shrubs and trees had similar PICs of force to punch (Fig. 4b; *P* = 0.356), LMA (Fig. 4c; *P* = 0.167), and leaf lifespan (Fig. 4d; *P* = 0.261). These results show that one of the four leaf traits varied significantly with life forms.

## Discussion

Although the use of SPAD values is now widespread^18^,^19^, no one has explicitly calibrated SPAD values with direct measurements of chlorophyll content across a broad range of plant species. Our results provide substantial evidence that SPAD values can serve as a good proxy of direct measurements of chlorophyll content at the species level. This result facilitates multiple species comparisons. Additionally, we found that leaf chlorophyll and root surface area were coordinated and that this coordination allowed plants to exhibit a high potential to yield biomass.

The most novel finding of our study was that leaf production potential and leaf tolerance potential were convergent across 107 woody species. This finding does not support the prediction of the worldwide leaf economics spectrum^7^,^8^, but it provides insights into the leaf economics spectrum at a given site. Actually, the photosynthetic capacity of leaves is not always negatively correlated with their structural and defensive costs. For example, tropical plants have a greater photosynthetic capacity and are better defended than temperate plants due to favourable resources and greater herbivore pressures in tropical habitats^20^,^21^,^22^. All the 107 species examined in this study have experienced the same climate and soil conditions since the 1980s, and all the measurements of leaf traits were completed within two weeks. Accordingly, the convergent leaf production and tolerance can be attributed primarily to long-term natural selection and genetic constraints.

Green leaves face two basic challenges, namely, carbon fixation and stress tolerance during their lifespan^1^,^9^,^15^, which can be incorporated into ecological strategies for balancing the cost of constructing a leaf versus the benefits that a leaf provides through carbon assimilation. The positive chlorophyll production relationship can stem from multiple causes. First, a high chlorophyll content tends to enable leaves to have high photosynthetic rates^11^,^17^, and LMA is positively correlated with maximum photosynthetic rates but negatively correlated with dark respiration^8^,^10^. Second, leaves with a high chlorophyll content have a greater LMA and are tougher, enabling them to be more resistant to abiotic and biotic stresses^9^,^12^,^15^,^16^. Finally, plants with a high chlorophyll content have a larger root surface area, enhancing their potential to absorb soil resources^23^. Taken together, these coordinated trait relations allow plants to maximize the total amount of carbon gain during their lifespan.

In the field, plants commonly face multiple stresses such as drought, temperature, and herbivory^3^. We found that leaf toughness and LMA increased with chlorophyll content. High leaf toughness and LMA help leaves have higher tolerance to multiple stresses by decreasing their vulnerability to stresses^7^,^8^,^9^,^12^,^15^,^16^,^17^,^18^,^19^,^20^,^21^,^22^,^23^,^24^. Evolutionary history and selective pressures contribute to this functional convergence through eliminating individuals with decoupled variation in production potential and multiple-stress tolerance and favouring individuals with coordinated variation in production potential and multiple-stress tolerance because they are key drivers of the evolution of the leaf economics spectrum^13^. Consequently, evolutionary history and natural selection shape convergent leaf trait relationships across 107 woody species.

LMA is positively correlated with leaf toughness^16^,^24^,^25^. Because LMA can be measured using simple and standardized procedures relative to leaf toughness, which is often determined using complex apparatus and approaches, LMA is a good proxy for leaf toughness. We found that neither LMA nor leaf toughness influenced leaf lifespan, a result contrary to previous reports^9^,^24^. This inconsistency is linked primarily to data analyses, that is, whether PICs are used or not. For example, LMA and leaf toughness affected leaf lifespan when these traits were analysed directly; in contrast, LMA and leaf toughness did not affect leaf lifespan when PICs were considered.

A second key finding of our study was that there was significant divergence in the leaf production potential between shrubs and trees. Specifically, shrubs had a higher leaf chlorophyll content than trees, but shrubs and trees had the same leaf lifespan, thereby allowing shrubs to have a greater potential to produce biomass. Shrubs and trees exhibited the same LMA and leaf toughness; thus, the palatability of leaves did not vary with functional types. Taken together, shrubs had a higher production potential but the same stress tolerance as trees. According to the worldwide leaf economics spectrum, leaf trait relationships may be independent of functional types^7^,^8^. However, our findings suggest that functional types may play an important role in shaping the leaf economics spectrum. This viewpoint is supported by previous findings that the differences in leaf size, N, and P occurred between shrubs and trees worldwide^26^. Our results also highlight that the legacies of natural selection matter in governing functional divergence between different functional types.

The patterns of leaf functional traits between shrubs and trees have several implications. For example, shrubs commonly experience poorer light resources relative to trees in nature. High levels of chlorophyll may be an adaptive strategy allowing shrubs to cope with a weak light environment. In contrast, trees have less chlorophyll due to richer light resources. Second, shrubs and trees had the same leaf toughness and LMA, exhibiting similar palatability. Third, leaf toughness determines its litter decomposition^9^,^18^,^27^, thus the leaves of shrubs and trees may share the same potential to return carbon and nutrients.

In summary, our findings suggest that convergent leaf production and stress tolerance may occur in woody plants and that divergent leaf production may appear between shrubs and trees. The generality of these patterns needs to be further tested across multiple sites. Area-based chlorophyll content values are likely to allow us to rapidly determine multiple leaf functions (e.g., production potential, stress tolerance, and carbon and nutrient returns) of intact plants in the field. More importantly, the chlorophyll-based trait spectrum might provide a useful basis for incorporating multiple functions into a framework.

## Methods

### Study garden

We conducted this study at the Botanical Garden of the Chinese Academy of Sciences (BGCAS: 39.98°N, 116.20°E, 80 m; close to the Fragrant Hills and 30 km from downtown Beijing). The BGCAS is located in a warm temperate region and is characterized by cinnamon soil (a type of soil), a mean annual temperature of 12 °C, and a mean annual precipitation of 500 mm. Our focal garden occupies a 300 × 200 m area (i.e., a smaller garden within the larger BGCAS) so that climate, parent material, hydrology, topography, and previous land use are relatively homogeneous. Since 1955, a number of different plant species across China have been transplanted to the BGCAS.

### Chlorophyll calibration

Although SPAD values have been widely used^18^,^19^, no study has explicitly calibrated the relations between SPAD values and direct measurements of the leaf chlorophyll content across a range of plant species. To do so, we randomly selected nine shrubs and nine trees from the species pools at the BGCAS (see Supplementary Table S1). We selected 3-5 fully developed leaves from each of the 10 individuals per species to measure their chlorophyll with SPAD-502 (Konica Minolta, Japan) in July 2012, six readings per leaf were recorded, and all readings per individual were averaged. We harvested these leaves and took them to the laboratory for directly measuring the leaf chlorophyll content. Chlorophyll was determined spectrophotometrically by measuring the absorbance of the extract at various wavelengths. Leaf punches were weighed and then placed in 10-mL centrifuge tubes with a 9-mL mixture of 95% ethanol and 80% acetone (v:v = 1:1). This extraction was stored for 24 h in the dark. Finally, we recorded the absorbance of each tube at A_663_ and A_645_. The total content of leaf chlorophyll a and b was calculated on the basis of leaf area and mass, respectively.

### Growth experiment

To quantify the relationships of SPAD values with root surface area and biomass production, we conducted an experiment because it is impossible to quantify these relationships in the field. All plants from seed were grown in 1-L pots filled with local soil. This experiment lasted five months from May to September 2013. Prior to harvest, we selected five leaves per plant for measuring their chlorophyll with SPAD-502. At the end of the experiment, all plants were harvested, and root surface area was determined by scanning with WinRHIZO/WinFOLIA (Regent Instruments, Canada). All the materials were oven-dried at 85°C for 48 h and then weighed. There were 30 replicates.

### Trait measurements *in situ*

All focal leaves met the following requirements: broad leaves and exposed to sunlight; the numbers of individuals per species were all greater than 10. Accordingly, 107 woody plant species were available in the common garden, including 60 tree species and 47 shrub species (totalling 35 families, see Supplementary Table S1). We excluded all other plant species that did not meet the above-mentioned criteria. We measured leaf chlorophyll, leaf toughness, and LMA in July 2010.

Five fully developed leaves from each of the 10 individuals per species were selected to measure chlorophyll with SPAD-502. All 10 readings per individual were averaged. We measured 107 different plant species. Each of these species was represented by 10 individuals.

We collected three fully expanded leaves from each of the 10 individuals per species and then sealed them in plastic bags to avoid a loss of turgor pressure. Each leaf was tested for mechanical strength using a general testing machine (5542, Instron, Canton, MA, USA). A flat-ended, sharp-edged cylindrical steel punch (diameter 1.2 mm) and a steel die with a sharp-edged hole with a small clearance (0.2 mm) were used. The punch and die were installed in the general testing machine, the punch was placed to pass through the middle of the hole of the die without any friction, and the punch speed was kept constant (20 mm min^−1^). This punch-and-die test was applied to randomly selected intercostal lamina (between secondary veins) for each leaf (two measurements per leaf). We measured 107 different plant species, each with 10 individuals.

After the measurements of force to punch, the areas of all the leaves were determined by scanning with WinRHIZO/WinFOLIA. All leaves were oven-dried at 75 °C for 48 h and then weighed. LMA was calculated by dividing the dry mass (g) by the leaf area (m^2^). We measured 107 different plant species, each with 10 individuals.

We determined leaf lifespan from a periodic census of tagged leaves. Because the measurements were labor-intensive, we measured the leaf lifespan in 56 selected species. In spring 2012, we tagged individual leaves when they unfolded for the first time and recorded whether they were alive or dead at two-week intervals. The tagged leaves were situated on four twigs on each of the nine individuals per species. Accordingly, we tagged 36 leaves per species at the beginning of this census. After the census, we calculated the lifespan for each individual leaf and took the average for each species. Due to conditions induced by artificial disturbances at the site, 49 species were available for leaf lifespan measurements, including 29 trees and 20 shrubs (see Supplementary Table S1).

### Data analyses

We attempted to test whether there was a correlation between SPAD values and direct measurements of the chlorophyll content across multiple species. For this purpose, we calculated the Pearson correlation coefficients. We found that SPAD values were correlated with the direct measurements of chlorophyll content based on leaf area but not leaf mass. Therefore, we selected area-based chlorophyll content as a variable for the following analyses.

The purpose of the controlled experiment was to quantify the three-way relationships among leaf chlorophyll, root surface area, and current-year production. Accordingly, we calculated the Pearson correlation coefficients to determine the relationship between the chlorophyll content and root surface area and how whole-plant biomass varied with chlorophyll content and root surface area.

To avoid non-independence among species, we calculated the PICs of leaf chlorophyll, force to punch, LMA, and leaf lifespan. Specifically, we created two plant phylogenies: one included 107 species for chlorophyll, LMA, and force to punch, and the other included 49 species for leaf lifespan. We created these two phylogenies using ‘Phylomatic’ software online (http://phylodiversity.net/phylomatic/). To resolve polytomies, randomization was performed with the ‘multi2di’ function in the ‘picante’ package^28^,^29^. The PICs were calculated using the ‘pic’ function in the ‘picante’ package (R3.0.1, R Development Core Team). To answer our first central question (i.e., relationships between leaf production potential and leaf stress tolerance potential across 107 woody species), we used correlation analyses to determine the three-way relationships of PICs of leaf chlorophyll with the PICs of LMA and force to punch. Additionally, we determined the relationship either between the PICs of LMA and the PICs of force to punch or between the PICs of leaf chlorophyll and the PICs of leaf lifespan using correlation analyses. This approach is valid for two reasons. LMA and force to punch are linked to the potential of leaves to tolerate stresses, and leaf chlorophyll and lifespan are associated with the potential of leaves to produce biomass.

To answer our second central question (i.e., comparisons of leaf production potential and leaf stress tolerance potential between shrubs and trees), we used a one-way analysis of variance to test the differences in PICs of leaf chlorophyll, force to punch, LMA, and leaf lifespan between shrubs and trees. All of these statistical analyses were performed with SPSS 13.0 (SPSS Inc.).

## Additional Information

**How to cite this article**: He, W.-M. and Sun, Z.-K. Convergent production and tolerance among 107 woody species and divergent production between shrubs and trees. *Sci. Rep.* **6**, 20485; doi: 10.1038/srep20485 (2016).

## Supplementary Material

Supplementary Information
