# Supplementary material for: Convergent production and tolerance among 107 woody species and divergent production between shrubs and trees
Source: Sci Rep. 2016 Feb 8;6:20485. doi: 10.1038/srep20485 (PMC4745073; doi:10.1038/srep20485)
Supplement: Supplementary Information [file srep20485-s1.pdf]

Convergent production and tolerance among 107 woody species and divergent production between shrubs and trees

Wei-Ming He & Zhen-Kai Sun

Supplementary Table S1: Species used in our study. Asterisks indicate that the corresponding traits were measured.

Species	Family	SPAD value	Leaf mass per area	Force to punch	Lifespan	Measured chlorophyll
**Trees**						
1. Acer truncatum	Aceraceae	*	*	*		
2. Ailanthus altissima	Simaroubaceae	*	*	*		
3. Carpinus betulus	Betulaceae	*	*	*		
4. Carpinus turczaninowii	Betulaceae	*	*	*	*	
5. Castanea mollissima	Fagaceae	*	*	*		
6. Catalpa ovate	Bignoniaceae	*	*	*	*	*
7. Celtis koraiersis	Ulmaceae	*	*	*		

8. Cercidiphyllum japonicum	Cercidiphyllaceae	*	*	*	*	
9. Chionanthus retusus	Oleaceae	*	*	*		
10. Cornus macrophylla	Cornaceae	*	*	*	*	
11. Cornus officinalis	Cornaceae	*	*	*	*	
12. Cornus walteri	Cornaceae	*	*	*	*	
13. Cronus japonica	Cornaceae	*	*	*		
14. Cudrania tricuspidata.	Moraceae	*	*	*		*
15. Dendrobenthamia japonica	Cornaceae	*	*	*		
16. Diospyros Iotus	Ebenaceae	*	*	*	*	
17. Eucommia ulmoides	Eucommiaceae	*	*	*	*	*
18. Euonymus bungeanus	Celastraceae	*	*	*	*	
19. Fraxinus bungeana	Oleaceae	*	*	*		
20. Fraxinus mandshurica	Oleaceae	*	*	*	*	*

21. Fraxinus paxiana	Oleaceae	*	*	*		
22. Fraxinus pennsylvanica	Oleaceae	*	*	*		
23. Fraxinus rhynchophylla	Oleaceae	*	*	*		
24. Gleditsia sinensis	Leguminosae	*	*	*	*	*
25. Ginkgo biloba	Ginkgoaceae	*	*	*	*	*
26. Hemiptelea davidii	Ulmaceae	*	*	*		
27. Juglans mandshurica	Juglandaceae	*	*	*	*	
28. Juglans regia	Juglandaceae	*	*	*	*	
29. Kalopanax septemlobus	Araliaceae	*	*	*	*	
30. Koelreuteria paniculata	Sapindaceae	*	*	*	*	
31. Liriodendron chinense	Magnoliaceae	*	*	*	*	
32. Magnolia biondii	Magnoliaceae	*	*	*		
33. Magnolia cylindrica	Magnoliaceae	*	*	*		

34. Magnolia liliflora	Magnoliaceae	*	*	*		
35. Magnolia soulangeana	Magnoliaceae	*	*	*	*	*
36. Magnolia zenii	Magnoliaceae	*	*	*		
37. Morus alba L.	Moraceae	*	*	*		
38. Prunus davidiana	Rosaceae	*	*	*		
39. Prunus mume	Rosaceae	*	*	*	*	
40. Prunus sargentii	Rosaceae	*	*	*		
41. Prunus yedoensis	Rosaceae	*	*	*	*	
42. Pteroceltis tatarinowii	Ulmaceae	*	*	*	*	*
43. Pyrus bretchneideri	Rosaceae	*	*	*	*	
44. Quercus acutissima	Fagaceae	*	*	*	*	*
45. Quercus aliena	Fagaceae	*	*	*	*	
46. Quercus liaotungensis	Fagaceae	*	*	*		

47. *Quercus serrata*	Fagaceae	*	*	*		
48. *Robinia pseudoacacia*	Leguminosae	*	*	*		
49. *Salix matsudana*	Salicaceae	*	*	*		
50. *Sophora japonica*	Leguminosae	*	*	*	*	
51. *Swida walteri*	Cornaceae	*	*	*		
52. *Tetradium ruticarpum*	Rutaceae	*	*	*	*	
53. *Tilia amurensis*	Tiliaceae	*	*	*		
54. *Ulmus castaneifolia*	Ulmaceae	*	*	*		
55. *Ulmus lamellosa*	Ulmaceae	*	*	*		
56. *Ulmus macrocarpa*	Ulmaceae	*	*	*		
57. *Ulmus parvifolia*	Ulmaceae	*	*	*		
58. *Xanthoceras sorbifolia*	Sapindaceae	*	*	*	*	
59. *Zelkova schneideriana*	Ulmaceae	*	*	*	*	

60. Zelkova serrata	Ulmaceae	*	*	*	*	
**Shrubs**						
61. Aralia chinensis	Araliaceae	*	*	*		
62. Berberis thunbergii	Berberidaceae	*	*	*	*	
63. Callicarpa bodinieri	Verbenaceae	*	*	*	*	*
64. Caragana sinica	Leguminosae	*	*	*	*	
65. Cercis chinensis	Leguminosae	*	*	*		
66. Chimonanthus praecox	Calycanthaceae	*	*	*	*	
67. Chimonanthus praecor	Calycanthaceae	*	*	*		
68. Corylus heterophylla	Betulaceae	*	*	*	*	*
69. Cotinus coggygria	Anacardiaceae	*	*	*	*	
70. Cotoneaster horizontalis	Rosaceae	*	*	*		

71. Deutzia grandiflora	Saxifragaceae	*	*	*	*	*
72. Elaeagnaceae pungens	Elaeagnaceae	*	*	*		
73. Elaeagnus umbellata	Elaeagnaceae	*	*	*		
74. Euonymus kiautschovicus	Celastraceae	*	*	*		
75. Forsythia suspensa	Oleaceae	*	*	*	*	*
76. Grewia biloba	Tiliaceae	*	*	*		
77. Hibiscus syriacus	Malvaceae	*	*	*	*	*
78. Jasminum nudiflorum	Oleaceae	*	*	*	*	
79. Kolkwitzia amabilis	Caprifoliaceae	*	*	*		
80. Lagerstroemia indica	Lythraceae	*	*	*	*	*
81. Ligustrum quihoui	Oleaceae	*	*	*		
82. Lonicera fragrantissima	Caprifoliaceae	*	*	*	*	*
83. Lonicera japonica	Caprifoliaceae	*	*	*		

84. Lonicera tricalysioides	Caprifoliaceae	*	*	*		
85. Philadelphus incanus	Saxifragaceae	*	*	*		
86. Philadelphus pekinensis	Saxifragaceae	*	*	*		
87. Prunus glandulosa	Rosaceae	*	*	*	*	
88. Rhamnus alaternus	Rhamnaceae	*	*	*	*	
89. Rhododendron micranthum	Ericaceae	*	*	*	*	
90. Rhododendron mucronulatum	Ericaceae	*	*	*	*	*
91. Rhodotypcs scandens	Rosaceae	*	*	*		
92. Rosa xanthina	Rosaceae	*	*	*		
93. Sambucus williamsii	Araliaceae	*	*	*	*	
94. Sorbaria kirilowii	Rosaceae	*	*	*		
95. Spiraea thunbergii	Rosaceae	*	*	*		
96. Syringa josikaea	Oleaceae	*	*	*		

97. Syringa meyeri	Oleaceae	*	*	*		
98. Syringa oblate	Oleaceae	*	*	*		
99. Syringa persica	Oleaceae	*	*	*		
100. Syringa velutina	Oleaceae	*	*	*		
101. Viburnum betulifolium	Caprifoliaceae	*	*	*		
102. Viburnum carlesii	Caprifoliaceae	*	*	*		
103. Viburnum farreri	Caprifoliaceae	*	*	*	*	*
104. Viburnum lantana	Caprifoliaceae	*	*	*	*	
105. Viburnum mongolicum	Caprifoliaceae	*	*	*		
106. Viburnum opulus	Caprifoliaceae	*	*	*	*	
107. Ziziphus jujube	Rhamnaceae	*	*	*		
